# Cost-effectiveness analysis of transcatheter aortic valve implantation versus surgical aortic valve replacement in patients with severe aortic stenosis at low risk of surgical mortality in Sweden

**DOI:** 10.48101/ujms.v130.10741

**Published:** 2025-04-04

**Authors:** Konrad Nilsson, Stefan James, Oskar Angerås, Jenny Backes, Henrik Bjursten, Pascal Candolfi, Mattias Götberg, Henrik Hagström, Chiara Malmberg, Niels Erik Nielsen, Archita Sarmah, Magnus Settergren, Tom Bromilow

**Affiliations:** a Department of Medical Sciences, Cardiology and Uppsala Clinical Research Center, Uppsala University, Uppsala, Sweden; b Department of Molecular and Clinical Medicine, Institute of Medicine, Gothenburg University, Gothenburg, Sweden; c Department of Cardiology, Sahlgrenska University Hospital, Gothenburg, Sweden; d Department of Cardiothoracic and Vascular Surgery, Örebro University Hospital, Örebro, Sweden; e Department of Cardiothoracic Surgery, Anaesthesia and Intensive Care, Lund University, Lund, Sweden; f Department of Cardiothoracic Surgery, Skåne University Hospital, Lund University, Lund, Sweden; g Edwards Lifesciences SA, Nyon, Switzerland; h Department of Cardiology, Skåne University Hospital, Clinical Sciences, Lund University, Lund, Sweden; i Department of Public Health and Clinical Medicine, Umeå University, Umeå, Sweden; j IHE – The Swedish Institute for Health Economics, Stockholm, Sweden; k Department of Cardiology, Heart Centre, University Hospital, Linköping, Sweden; l Heart and Vascular Unit, Karolinska University Hospital, Stockholm, Sweden; m Department of Medicine, Karolinska Institutet, Stockholm, Sweden; n York Health Economics Consortium, University of York, York, UK

**Keywords:** Transcatheter aortic valve implantation, surgical aortic valve replacement, cost-effectiveness, aortic stenosis, low risk

## Abstract

**Background:**

Transcatheter aortic valve implantation (TAVI) has shown similar or improved clinical outcomes compared with surgical aortic valve replacement (SAVR) in patients with symptomatic severe aortic stenosis at low risk for surgical mortality. This cost-utility analysis compared TAVI with SAPIEN 3 versus SAVR in symptomatic severe aortic stenosis patients at low risk of surgical mortality from the perspective of the Swedish healthcare system.

**Methods:**

A published, two-stage, Markov-based cost-utility model that captured clinical outcomes from the *Swedish Web-system for Enhancement and Development of Evidence-based care in Heart disease Evaluated according to Recommended Therapies* (SWEDEHEART) registry (2018–2020) was adapted from the perspective of the Swedish healthcare system using local general population mortality, utility and costs data. The model had a lifetime horizon. Model outputs included changes in direct healthcare costs and health-related quality of life from using TAVI as compared with SAVR.

**Results:**

TAVI with SAPIEN 3 resulted in lifetime costs per patient of 484,142 SEK Swedish krona (SEK) and lifetime quality-adjusted life years (QALYs) per patient of 7.16, whilst SAVR resulted in lifetime costs and QALYs per patient of 457,625 SEK and 6.81 QALYs, respectively. Compared with SAVR, TAVI offered an incremental improvement of +0.35 QALY per patient at an increased cost of +26,517 SEK per patient over a lifetime horizon, resulting in an incremental cost-effectiveness ratio of 76,532 SEK per QALY gained.

**Conclusion:**

TAVI with SAPIEN 3 is a cost-effective option versus SAVR for patients with symptomatic severe aortic stenosis at low risk for surgical mortality treated in the Swedish healthcare setting. These findings may inform policy decisions in Sweden for the management of this patient group.

## Introduction

Transcatheter aortic valve implantation (TAVI) has emerged as the treatment of choice over surgical aortic valve replacement (SAVR) for patients with severe symptomatic aortic stenosis (sSAS) at intermediate- and high-surgical risk (1–6). As technology has evolved, improvements in patients’ outcomes, quality of life and reduced complication rates have been reported, leading to TAVI also being used in low-risk patients (7).

The Placement of Aortic Transcatheter Valves (PARTNER) 3 trial was a multicentre, randomised controlled study, which demonstrated that TAVI, using the SAPIEN 3 valve, provided meaningful benefits in patients with sSAS, who were considered at low risk of surgical mortality compared with SAVR (8, 9). In addition to a significant reduction in the composite outcome of death, stroke or rehospitalisation at 1 and 2 years, TAVI was also associated with significantly lower rates of stroke and new-onset atrial fibrillation (AF), shorter index hospitalisation, higher functional status and improved quality of life at 30-days compared with SAVR (9). Furthermore, there were no significant differences between the groups in major vascular complications, new permanent pacemaker implantations or moderate or severe paravalvular regurgitation (8, 9). Finally, in the recently published 5-year results, event rates for death, stroke or rehospitalisation remained low and very similar to the surgical arm (10). This expanding evidence base allows TAVI to be considered for patients at high (11), intermediate (12) and, increasingly, low risk of surgical mortality (13).

The updated 2021 European Society of Cardiology/European Association for Cardio-Thoracic Surgery guidelines recommend TAVI in all patients aged ≥75 years with sSAS regardless of surgical risk status (recommendation class IA), providing there are no clinical or anatomical barriers and suitable femoral access (14).

Given the adoption of TAVI into various treatment guidelines, the demonstrated clinical benefits for patients, the high numbers of procedures performed to date and the potential for further adoption with the move towards use in lower risk patients, it is important to evaluate the implications on healthcare resources of potentially expanded TAVI use to inform clinicians and policymakers. To date, cost-effectiveness analyses have been published for France (15), Italy (16), Spain (17), Germany (18) and Belgium (19) that showed economic dominance or cost-effectiveness of TAVI with SAPIEN 3 versus SAVR in patients with sSAS at low surgical risk. These analyses used most of the clinical outcomes from the PARTNER 3 trial (9) and not from complete national clinical registries. Therefore, we conducted a cost-utility analysis using data from the *Swedish Web-system for Enhancement and Development of Evidence-based care in Heart disease Evaluated according to Recommended Therapies* (SWEDEHEART) registry (6) in combination with cost data from Sweden to adapt this Markov model and assess the value of TAVI with SAPIEN 3 versus SAVR in Swedish patients with sSAS at low risk of surgical mortality.

## Methods

### Model structure

A cost-utility analysis was conducted using methodology validated in previously published studies (15–19) to estimate changes in both direct healthcare costs and health-related quality of life with the use of TAVI with SAPIEN 3 versus isolated SAVR with any biological valve in sSAS patients at low risk of surgical mortality (as defined by a EuroSCORE II score <4%) from the perspective of the Swedish national healthcare system. For both TAVI and SAVR, patients between 70 and 80 years of age who received a registered procedure between 2018 and 2020 were included. Ethical approval of using Swedish registry data was granted (dnr 2017/455).

**Figure 1:**
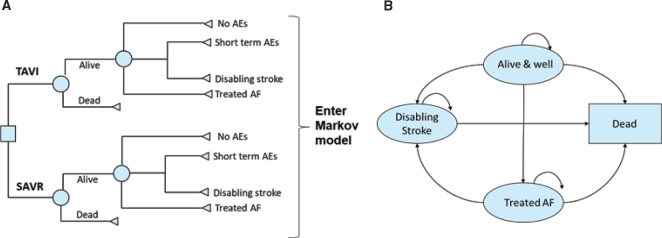
The cost-effectiveness model had two stages: **(A)** early AEs from the PARTNER 3 trial were captured in a decision tree, which fed into **(B)** a Markov model that captured longer-term outcomes of patients, with four distinct health states.^a^ Reproduced from Gilard M, et al. (15); https://doi.org/10.1016/j.jval.2021.10.003 under the terms of the creative commons licence (Creative Commons Attribution License (CC BY)). ^a^‘Alive and well’: patients have undergone the procedure and survived with only short-term or no AEs; patients in this health state can transition to ‘disabling stroke’, ‘AF’ or ‘dead’ at any point during the model time horizon. ‘Treated AF’: patients have undergone the procedure and survived but developed AF requiring specific treatment; this can either occur within the first 30 days or during the rest of the time horizon of the model, and patients in this health state can transition to ‘disabling stroke’ or ‘dead’ at any point during the model time horizon. ‘Disabling stroke’: patients have undergone the procedure and survived but had a disabling stroke; this can either occur within the first 30 days or during the rest of the time horizon of the model, and patients in this health state can only transition into the ‘dead’ state at any point during the model time horizon. ‘Dead’: this is the absorbing state in the model: all patients in the model are at risk of dying due to general all-cause mortality; patients with treated AF and stroke are at an increased risk of dying. AE, adverse event; AF, atrial fibrillation; SAVR, surgical aortic valve replacement; TAVI, transcatheter aortic valve implantation.

A two-stage model structure was used to form the basis of the cost-utility analysis, details of which have been published previously (15). In brief, early adverse events (AEs) linked to each procedure were captured primarily from the SWEDEHEART registry (6) and entered into a decision tree (Figure 1a) (15). Data on three events were not available from the registry and were obtained from the PARTNER 3 trial (9). The decision tree outcomes were subsequently fed into a Markov model, which included four distinct health states (‘Alive and well’, ‘Atrial fibrillation’, ‘Disabling stroke’ and ‘Dead’), to capture longer-term patient outcomes post-TAVI or SAVR intervention (Figure 1b) (15). Markov models are the most common model type used for economic evaluations of healthcare interventions (quantifying the costs and health benefits associated with an intervention) (20). These models are cohort-based and capture disease progression via transitions between mutually exclusive health states over discrete time periods (cycles). Their main strength is that they are simple and extremely adaptable, which is shown by their use across many clinical indications (21). For those unfamiliar with economic evaluation, a glossary of key terms can be found here: www.yhec.co.uk/glossary/ and a reader’s guide to facilitate reading and interpretation is detailed in Abbott et al. (22) The model structure was validated for the Swedish context by the authors, based on their clinical and health-economic expertise.

Considering that sSAS requires life-long valve replacement, a lifetime horizon was selected to reflect all possible consequences to individuals with sSAS over their lifetime, and a discounting factor per year of 3% was applied for both future costs and benefits (23).

Health-related quality of life was included in the analysis using quality-adjusted life years (QALYs) as an endpoint with EuroQol-5 Dimensions (EQ-5D) questionnaire utility decrements for the AF and stroke health states taken from the published literature and adjusted for age and population norms using Burström et al. (24).

### Model inputs

#### Clinical events

In the base case, data on clinical events within 1 month after the procedure were extracted for the SAPIEN 3 (from a total of 603 procedures, 204 involved patients aged 70–80 years and logistic EuroSCORE <4% were included) and the SAVR (*n* = 1,375) groups from the SWEDEHEART sub-registers for TAVI and SAVR, the Swedish Transcatheter Cardiac Intervention Registry and the Swedish Cardiac Surgery Registry, respectively. With all Swedish centres (TAVI = 8 centres; SAVR = 8 centres) contributing data to the registries, they have complete national coverage. The registries hold information on clinical patient characteristics, echocardiographic findings, procedures and periprocedural outcomes. Details on the registries have been described in earlier publications (25, 26). For a few outcomes not covered by the registries, data were collected from the National Patient Registry using the International Classification of Disease – 10th revision (ICD-10) codes. Stroke was defined as an ICD-10 code starting with I63, and, thus, it was not possible to grade the severity of disability. For the remaining four outcomes (one for the TAVI arm and three for the SAVR arm), where data were not available in either of the registries, PARTNER 3 (9) outcomes were used (Supplementary Table 1). Data from the registry were also used to estimate the monthly probability of transitioning from ‘Alive and well’ to ‘Treated AF’, from ‘Alive and well’ to ‘Disabling stroke’ and from ‘Treated AF’ to ‘Disabling stroke’. Furthermore, data on the probability of aortic reintervention due to valve deterioration as well as rehospitalisation rates up to 3 years (post which rates were assumed to remain constant over the remaining time horizon of the model) were also obtained from the registry. For the TAVI arm, data were additionally extracted for the pooled SAPIEN 3 and SAPIEN 3 ultra sample (*n* = 373) for use within a scenario analysis.

The annual mortality risk for ‘alive and well’ by gender was obtained from National Life Tables Sweden (2022) (27) (Supplementary Table 2). The relative risk of death (hazard ratio [HR]) associated with being in the treated AF or disabling stroke health states was obtained from Odutayo et al. (28) and Dennis et al. (29), whilst the relative risk of undergoing an aortic reintervention was sourced from PARTNER 3 (9).

#### Cost inputs

The cost perspective was based on information from the Swedish Diagnosis Related Group (DRG) tariffs and from published literature (Table 1). Costs were indexed to 2022 unless otherwise stated. For the base case, costs associated with TAVI and SAVR procedures were estimated from the DRG tariffs and published literature (Table 2). Costs for rehabilitation following TAVI and SAVR were based on the Swedish DRG tariffs and from published literature, indexed to 2022. Costs associated with health states, 30-days AEs (myocardial infarction, non-disabling stroke, transient ischaemic attack, bleeding and acute kidney injury), intercurrent events (myocardial infarction, transient ischaemic attack and bleeding), rehospitalisation and pacemaker implantation were estimated from DRGs and/or published literature (Tables 1 and 2). Reintervention costs were assumed to be equal to the combined costs of the initial procedure and rehabilitation associated with the procedure.

**Table 1:** Costs associated with TAVI and SAVR (procedure, complications and long-term).

Unit cost components	TAVI with SAPIEN 3	SAVR	Source
Procedure	302,329 SEK	244,750 SEK	Swedish Health Care Regions (2022) – Average of DRGs E04E and E03N across price lists for all regions
**Acute post-operative complications**			
Reintervention	302,329 SEK		Assumed equal to cost of initial procedure plus rehabilitation associated with procedure
**Associated to health states**			
Treated AF – Month 1	55,561 SEK		Hallberg et al. (30)
Treated AF ≥ Month 2	2,111 SEK		
Disabling stroke – Month 1	106,165 SEK		Lanitis et al. (31)
Disabling stroke ≥ Month 2	4,284 SEK		
Caregiver for disabling stroke – Month 1	69,491 SEK		
Caregiver for disabling stroke ≥ Month 2	37,970 SEK		
Alive and well – Year 1 (cost per month)	685 SEK		Swedish Health Care Regions (2022) – Average of ‘besök kardiolog’ across price lists for all regions (assumed 2 visits in Year 1 and 1 visit Year 2+)
Alive and well – Year 2+ (cost per month)	342 SEK		
**Other costs considered**			
Pacemaker procedure	77,451 SEK		Swedish Health Care Regions (2022) – Average of E26A, E26C and E26E across price lists for all regions
Pacemaker complications (monthly)	3,206 SEK		Swedish Health Care Regions (2022) – Average of X31O across price lists for all regions.
Rehospitalisation	61,340 SEK		Swedish Health Care Regions (2022) – Average of E47A, E47C and E47E across price lists for all regions

AF, atrial fibrillation; SAVR, surgical aortic valve replacement; TAVI, transcatheter aortic valve implantation.

**Table 2:** Breakdown of TAVI and SAVR procedure costs.

	TAVI with SAPIEN 3	SAVR	Source
**Procedure**	301,079 SEK	244,301 SEK	Swedish Health Care Regions (2022) – Average of E03N (TAVI) and E04E (SAVR) across price lists for all regions
Rehabilitation	3,973 SEK	3,973 SEK	Wittboldt et al. (32)
Rehabilitation rate	2.8%	11.3%	PARTNER 3 (9)
**Rehabilitation**	111 SEK	449 SEK	
Pacemaker insertion	77,451 SEK	77,451 SEK	Swedish Health Care Regions (2022) – Average of E26A, E26C and E26E across price lists for all regions
Permanent pacemaker insertion rate	1.5%	0%	SWEDEHEART registry (2018–2020) (6)
**Pacemaker insertion**	1,139 SEK	0 SEK	
**Total procedure costs**	**302,329 SEK**	**244,750 SEK**	

SAVR, surgical aortic valve replacement; TAVI, transcatheter aortic valve implantation.

#### Utilities

Age-adjusted population utility norms were used. An EQ-5D index value was used to document the population utility scores by age group (24) specific to the Swedish population. The small number of events in the PARTNER 3 trial meant that utility decrements were estimated using the published literature. Disabling stroke disutility was estimated based on a published study (31), whilst disutility for AF was estimated from Ref. (33). Disutility data were not included for intercurrent events in order to avoid a risk of double counting with the health state utilities applied to patients in the ‘treated AF’ and ‘disabling stroke’ states.

### Model outputs

Details of the model outputs and assumptions have been published previously (15). All analyses were performed using Microsoft Excel (Microsoft Corporation, Redmond, WA). The model generated total per-patient costs and QALYs for each intervention over the patients’ lifetime, and an incremental cost-effectiveness ratio (ICER) for TAVI with SAPIEN 3 versus SAVR in Swedish low-risk patients with sSAS. In Sweden, there is no official willingness-to-pay (WTP) threshold. However, for the Tandvårds- och läkemedelsförmånsverket or The Swedish Dental and Pharmaceutical Benefits Agency that makes reimbursement decisions for prescription drugs as well as reviews cost-effectiveness analysis for the Council on New Therapies, the unofficial WTP threshold is 1,000,000 SEK for treatments of very high severity diseases (34). This is the WTP threshold assumed in this study, and it is based on published literature.

#### Sensitivity and scenario analyses

To evaluate uncertainty, univariate deterministic sensitivity analyses (DSAs) were performed by varying inputs using confidence intervals and ranges from the literature when available, and plausible ranges when data were unavailable (Supplementary Table 3). All parameters were changed, and the impact on the results explored. Overall parameter uncertainty was addressed using a probabilistic sensitivity analysis (PSA). Probability distributions for all input parameters were specified, and 1,000 Monte Carlo simulations were run using random draws of all parameters from within their assigned distributions (Supplementary Table 4). In addition, 16 scenario analyses were run to test a number of assumptions on the clinical inputs, time horizon, the discount factor and the survival data. Of these, three scenarios specially addressed the issues of development of postoperative AF and the use of non-vitamin K antagonist anticoagulants in SAVR patients. Scenario 13 considered a relative risk of mortality with treated AF of 1 to remove any survival benefit with TAVI. Scenario 14 assumed zero monthly costs associated with the health state of AF to avoid attributing a cost to SAVR not witnessed in clinical practice. Finally, Scenario 15 used late postoperative AF data and a HR of 5.1 for mortality associated with AF from PARTNER 3 (35).

## Results

### Base case

Compared with SAVR, TAVI is estimated to offer an incremental health benefit of +0.35 QALYs per patient at an incremental cost of +26,517 SEK per patient over a lifetime horizon. This represents an ICER of 76,532 SEK per QALY gained (Table 3). Overall, TAVI with SAPIEN 3 resulted in lifetime costs per patient of 484,142 SEK and lifetime QALY per patient of 7.16, and for SAVR, these values were 457,625 SEK and 6.81 QALYs, respectively (Table 3).

**Table 3:** Base case results with acute and lifetime costs.

Summary results	TAVI with SAPIEN 3	SAVR	Incremental
**Cost per patient**	**484,142 SEK**	**457,625 SEK**	**26,517 SEK**
Life year gained (undiscounted)	11.79	11.36	0.43
Median survival (years)	13.75	12.00	1.75
**QALYs per patient**	**7.16**	**6.81**	**0.35**
**Incremental cost effectiveness ratio (ICER)**			**76,532 SEK**
**Incremental net monetary benefit (NMB)**			319,964 SEK
**Incremental net health benefit (NHB)**			0.32
*Acute phase cost (first hospitalisation and rehabilitation)*			
Index hospitalisation	302,218 SEK	244,301 SEK	57,917 SEK
Rehabilitation *	111 SEK	449 SEK	−338 SEK
**Acute phase costs**	**302,329 SEK**	**244,750 SEK**	57,579 SEK
*Additional costs at 1 year*			
MI	0 SEK	525 SEK	−525 SEK
Costs of pacemaker complications	500 SEK	0 SEK	500 SEK
Costs of hospitalizations	30,130 SEK	40,427 SEK	−10,297 SEK
Re-intervention costs	192 SEK	191 SEK	1 SEK
Alive & well health state costs	7,654 SEK	6,120 SEK	1,534 SEK
Treated AF health state costs	2,195 SEK	16,975 SEK	−14,780 SEK
Disabling stroke costs	10,347 SEK	9,393 SEK	955 SEK
Death costs	774 SEK	932 SEK	−158 SEK
**Total costs at 1 year**	**354,121 SEK**	**319,313 SEK**	34,808 SEK
*Additional lifetime costs*			
Costs of pacemaker complications	4,863 SEK	0 SEK	4,863 SEK
Costs of hospitalizations	9,826 SEK	4,019 SEK	5,807 SEK
Re-intervention costs	1,783 SEK	1,722 SEK	61 SEK
Alive and well health state costs	33,833 SEK	26,945 SEK	6,888 SEK
Treated AF health state costs	6,337 SEK	41,783 SEK	−35,445 SEK
Disabling stroke costs	56,770 SEK	47,178 SEK	9,592 SEK
Death costs	16,609 SEK	16,666 SEK	−57 SEK
**Additional lifetime costs**	130,021 SEK	138,312 SEK	−8,291 SEK
**Total lifetime costs**	**484,142 SEK**	**457,625 SEK**	**26,517 SEK**

SAVR, surgical aortic valve replacement; TAVI, transcatheter aortic valve implantation; AF, atrial fibrillation; MI, myocardial infarction; QALY, quality-adjusted life years.

**Figure 2:**
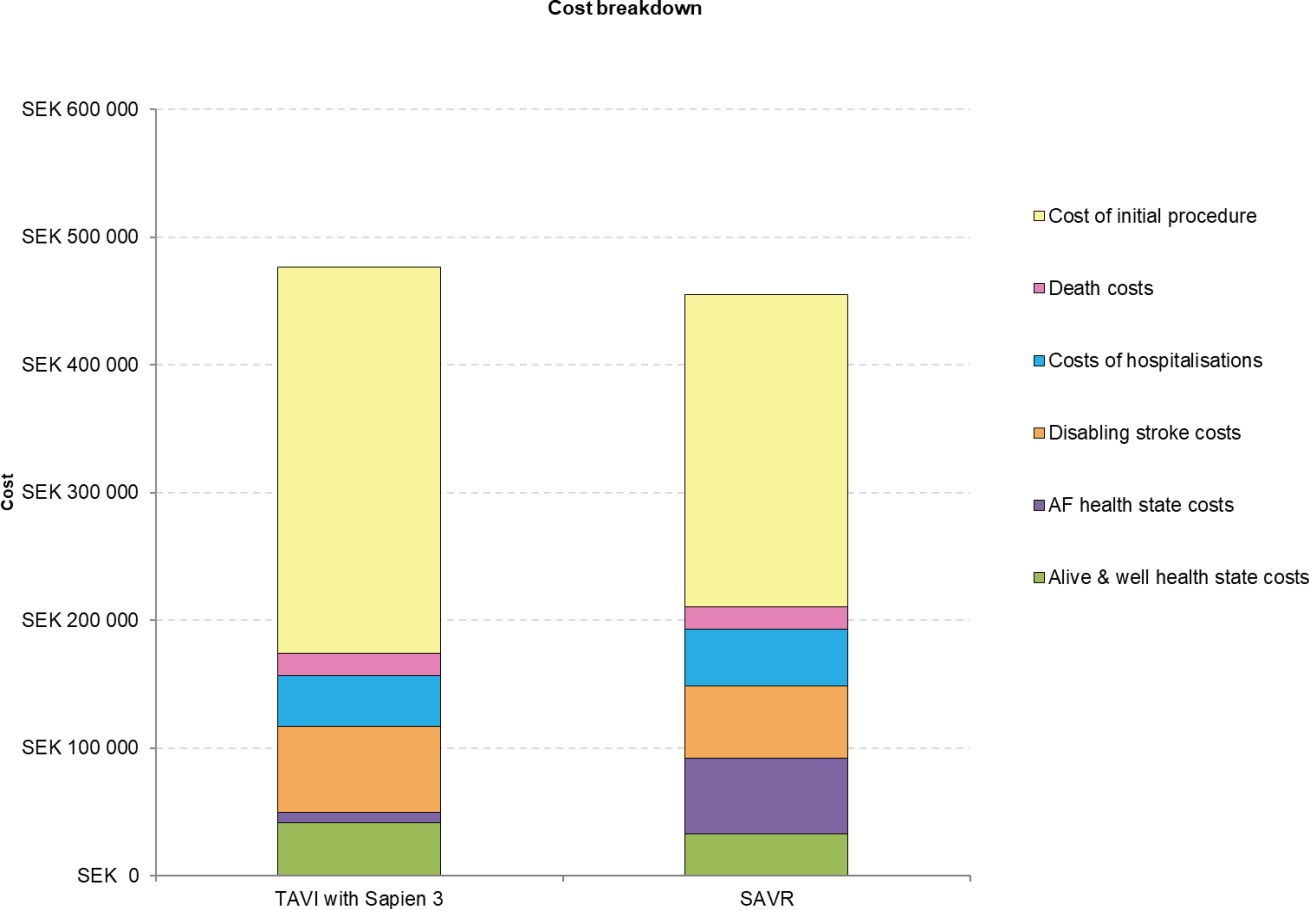
Cost breakdown for TAVI with SAPIEN 3 and for SAVR. AF, atrial fibrillation; SAVR, surgical aortic valve replacement; TAVI, transcatheter aortic valve implantation.

A detailed breakdown of costs revealed higher acute phase costs, alive and well health state costs, and disabling stroke costs for TAVI with SAPIEN 3 versus SAVR, but lower costs for treated AF and hospitalisation (Table 3; Figure 2).

### Sensitivity analyses

**Figure 3:**
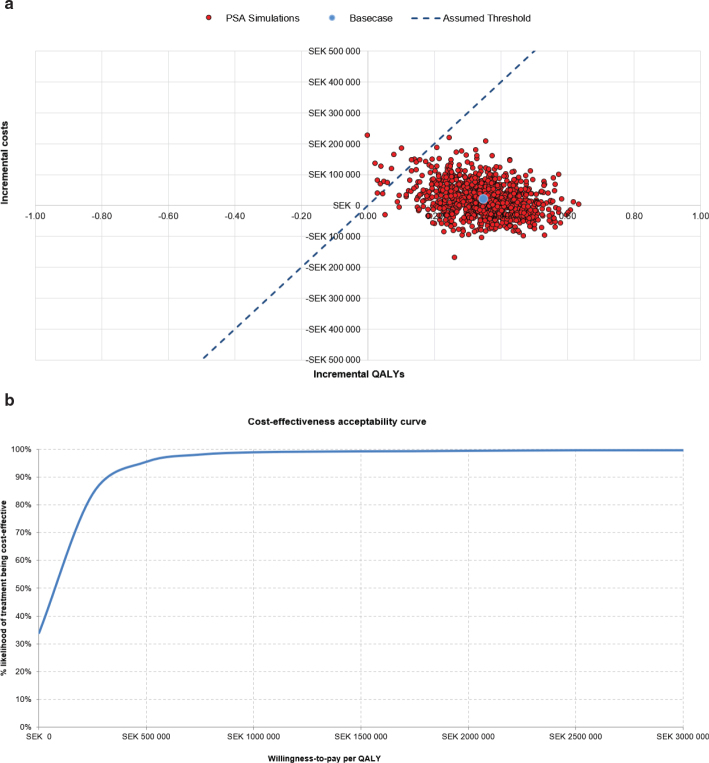
Probabilistic sensitivity analysis: **(a)** cost-effectiveness scatter plot and **(b)** cost-effectiveness acceptability curve.

The findings of the PSA corroborate those of the base case analysis. At the assumed Swedish WTP threshold of 1,000,000 SEK/QALY, TAVI with SAPIEN 3 had an 99% probability of being cost-effective (Figure 3a). Assuming a more conservative cost-effectiveness threshold of 500,000 SEK/QALY, there is still a 96% likelihood of TAVI with SAPIEN 3 being cost-effective compared to SAVR (Figure 3b).

**Figure 4:**
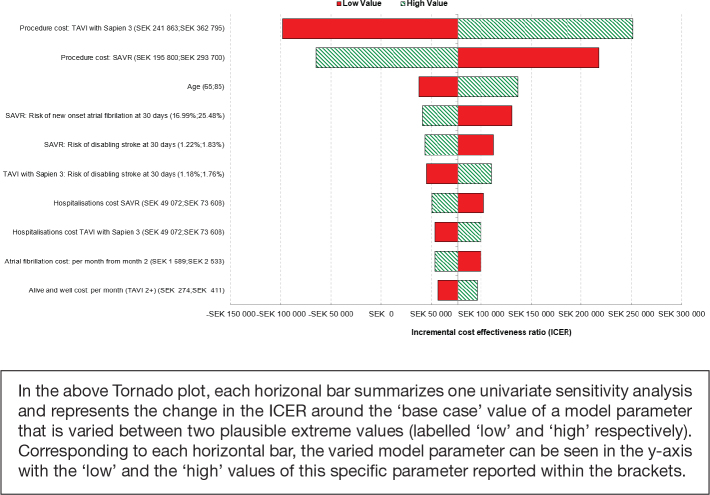
Tornado diagram showing the 10 parameters with greatest influence on the model (deterministic sensitivity analysis). AF, atrial fibrillation; SAVR, surgical aortic valve replacement; TAVI, transcatheter aortic valve implantation.

Univariate DSA demonstrated that TAVI with SAPIEN 3 remained cost-effective, compared with SAVR, regardless of plausible changes in individual model parameters (Figure 4). The model was most sensitive to procedure costs of TAVI with SAPIEN 3 and procedure costs for SAVR (Figure 4).

The results from the various scenario analyses demonstrated the comparative robustness of the model reported and are presented in Table 4.

**Table 4:** Scenario analyses results.

Scenarios		Incremental costs (TAVI vs SAVR)	Incremental QALYs (TAVI vs SAVR)	ICER (Incremental cost effectiveness ratio)
**Base case**		26,517 SEK	0.35	76,532 SEK/QALY
Scenario 1	Survival data from PARTNER 3	40,809 SEK	0.97	41,917 SEK/QALY
Scenario 2	No survival benefit with TAVI	24,550 SEK	0.18	139,197 SEK/QALY
Scenario 3	Including AE costs within 30 days	15,504 SEK	0.35	44,748 SEK/QALY
Scenario 4	Time horizon = 5 years	34,422 SEK	0.10	355,137 SEK/QALY
Scenario 5	Time horizon = 10 years	29,075 SEK	0.20	148,456 SEK/QALY
Scenario 6	Time horizon = 15 years	26,654 SEK	0.28	94,507 SEK/QALY
Scenario 7	Time horizon = 20 years	26,331 SEK	0.33	79,543 SEK/QALY
Scenario 8	Time horizon = 30 years	26,513 SEK	0.35	76,554 SEK/QALY
Scenario 9	Clinical inputs from SWEDEHEART registry data (2018–2020) for SAPIEN 3 and SAPIEN 3 ultra pooled sample	29,113 SEK	0.38	76,573 SEK/QALY
Scenario 10	Costs and outcomes both discounted at 0%	24,227 SEK	0.46	52,300 SEK/QALY
Scenario 11	Costs and outcomes both discounted at 5%	27,694 SEK	0.29	94,986 SEK/QALY
Scenario 12	Costs discounted at 3% and outcomes at 0%	26,517 SEK	0.46	57,243 SEK/QALY
Scenario 13	RR of death with treated AF= 1	19,981 SEK	0.17	114,716 SEK/QALY
Scenario 14	Monthly costs associated with treated AF health state= 0	76,743 SEK	0.35	221,492 SEK/QALY
Scenario 15	Using late POAF data from PARTNER 3	60,570 SEK	0.30	204,901 SEK/QALY
Scenario 16	Using 2022 values from KPP database for TAVI (277,327 SEK) and SAVR (243,462 SEK)	3,599 SEK	0.35	10,387 SEK/QALY

SAVR, surgical aortic valve replacement; TAVI, transcatheter aortic valve implantation; AF, atrial fibrillation; POAF, postoperative atrial fibrillation; RR, relative risk, KPP, Kostnad per patient (cost per patient.

## Discussion

This analysis using real-world clinical outcomes from Sweden indicates that TAVI with SAPIEN 3 is expected to be a cost-effective valve replacement alternative versus SAVR for Swedish low surgical risk sSAS patients. The ICER of 76,532 SEK per QALY gained is within the assumed WTP threshold of 1,000,000 SEK per QALY. The incremental QALY gain of +0.35 is also within the range of those reported in previously published cost-effectiveness evaluations of TAVI and of pharmaceutical interventions in cardiology (36–38). Sensitivity analyses were used to assess uncertainty, and the results appeared robust.

A detailed breakdown of costs revealed higher acute phase costs, ‘alive and well’ health state costs, and disabling stroke costs for TAVI with SAPIEN 3 versus SAVR, but lower costs for treated AF and hospitalisation. Initial procedure costs for TAVI with SAPIEN 3 were higher than for SAVR in Sweden, which was also the case for Italy (16), Spain (17), Germany (18) and Belgium (19), whereas the initial cost for performing TAVI was lower than for SAVR in France (15). It is worth noting though that data from the registry show that the length of stay for TAVI is shorter as compared with SAVR (mean of 3 days for TAVI versus 8 days for SAVR). By implication, this should result in lower procedural costs for TAVI in comparison to that for SAVR. This corroborates our results from the base case that took a conservative approach of using DRG values for both the procedures.

These findings are consistent with others reported for TAVI with SAPIEN 3 versus SAVR in other countries in the European Union. For example, TAVI with SAPIEN 3 was shown to be dominant over SAVR in low-risk patients with sSAS in France (15, 39) and in Belgium (19) and was cost-effective over SAVR in Italy (16), Spain (17) and Germany (18). TAVI with SAPIEN 3 has also been reported to be dominant over SAVR in low-risk patients in Norway (40) and Ireland (41), and cost-effective compared with SAVR in Australia (42) and Canada (43).

Patients tend to prefer minimally invasive interventions as they are usually associated with less discomfort, lower risk of complications and/or rehospitalisation (44). The clinical benefits of TAVI with SAPIEN 3 in low-risk patients with sSAS have been established in PARTNER 3, which reported a lower risk of infection, fewer complications and shorter hospital stays, compared with SAVR, whilst also improving patients’ quality of life (8, 9).

From the healthcare provider perspective, TAVI provides efficiencies by reducing healthcare resource use, post-surgical complications and hospital stays. Reducing hospital stays allows higher patient intake capacity, which is an important consideration for health systems under high demand and with long waiting lists. TAVI presents benefits by reducing the recovery period to resuming normal activity that might not be accounted for in this analysis. Further indirect benefits may include a reduced need for caregiver support.

### Limitations

This study comes with certain limitations. First, using data from registers comes with inherited weaknesses such as dependency on correct entries and on factors built into the registry. However, for both SWEDEHEART and the National Patient Register, high levels of data agreement have been reported (6, 45).

Second, the results cannot be generalised to all patients with aortic stenosis as the PARTNER 3 trial excluded patients with clinical frailty, bicuspid aortic valves or other unsuitable anatomical features that increased the risk of complications post-intervention. Caution must also be exercised when attempting to generalise the findings from this model to populations outside Sweden.

Third, in the base case, we made the conservative choice to use the average costs of DRG tariffs for the Swedish healthcare regions. This tariff is not specific to low-risk symptomatic severe aortic stenosis patients, and it is likely that it is overestimated for a low-risk TAVI patient treated under current practice. To account nevertheless for the uncertainty introduced with this choice, we used the alternative cost estimates for TAVI and SAVR procedures available from the Kostnad per patient (KPP) database (46) within a scenario analysis. It is worth noting that unlike the region’s price lists, which reflect current costs, the costs from the KPP database depend on the costing levels of the past years and reflect the costs of the entire intervention process. The results corroborate the findings of the base case.

Fourth, it is worth noting that there are inherent limitations in any cost-effectiveness analysis including: assumptions made in the presence of ‘best fit’ data or paucity of data; extrapolations modelled for time horizons beyond the scope of existing input data; and the potential for under- and over-estimations as a result of differences in healthcare systems and/or the intervention and treatment selection criteria within a specific system.

Finally, the cost-effectiveness findings from this study cannot be generalised to other TAVI devices beyond the SAPIEN 3 device.

## Conclusion

In this analysis, leveraging real-world evidence from the SWEDEHEART registry, TAVI with SAPIEN 3 is likely to offer a cost-effective intervention versus SAVR in low-risk patients with sSAS in Sweden. These data may be helpful for informing clinicians, policymakers and healthcare budget holders in Sweden in their planning on how best to optimise both health outcomes and resource allocation in the management of patients with sSAS.

## Supplementary Material



## Data Availability

The datasets used in this article are legally restricted because of Swedish privacy and secrecy laws and are therefore not publicly available.
